# Freckles

**Published:** 1878-06

**Authors:** 


Freckles.—Take of finely powdered sulphophenate of zinc, one part; oil of lemon, one part; pure alcohol, five parts; collodion, forty-five parts; mix well together by trituration. This has been found efficacious as a local application against freckles and other slight skin diseases.—*Pharm. Zeit. fur Russ.*

